# The role of body composition and visceral fat in osteoporosis subtype differentiation: Insights from bioelectrical impedance analysis

**DOI:** 10.1016/j.jor.2025.06.014

**Published:** 2025-06-18

**Authors:** N. Wergen, U. Maus, K. Schultz, H. Frohnhofen, D. Latz, C. Somsen, L. Mueller, C. Beyersdorf

**Affiliations:** a Department for Orthopedics and Trauma Surgery, Medical Faculty, University Hospital Düsseldorf, Heinrich Heine University Düsseldorf, 40225, Duesseldorf, Germany; b Heinrich Heine University Düsseldorf, 40225, Duesseldorf, Germany

**Keywords:** Osteoporosis, BIA, Visceral fat, Inflammation

## Abstract

**Background:**

Recent studies have demonstrated a close link between body composition and the development and progression of osteoporosis. Visceral fat, in particular, appears to influence bone loss through its pro-inflammatory properties. However, it remains unclear whether this mechanism is equally relevant across different forms of osteoporosis.

**Objective:**

To investigate whether body composition—especially visceral fat— differs between postmenopausal and senile osteoporosis.

**Participants and setting:**

A total of 47 patients were prospectively enrolled. The senile osteoporosis group included patients aged ≥80 years (n = 20, mean age 87.4), the postmenopausal osteoporosis group included patients aged ≤75 years (n = 14, mean age 68.8), and the control group consisted of patients aged ≤75 years (n = 13, mean age 68.8) without osteoporotic fractures or other osteoporosis-specific risk factors.

**Methods:**

Participants underwent bioelectrical impedance analysis (BIA) to assess body composition. Additional assessments included basic osteological laboratory testing, geriatric evaluation, sarcopenia screening (SARC-F), and frailty screening using the Clinical Frailty Scale (CFS).

**Results:**

Muscle mass, total body water, fat-free mass, and BMI were significantly reduced in the senile osteoporosis group compared to controls. Similar trends were observed in the postmenopausal group, though without statistical significance. Notably, the senile osteoporosis group had a significantly higher proportion of visceral fat relative to total fat mass than both the control and postmenopausal groups.

**Conclusion:**

Patients with senile and postmenopausal osteoporosis exhibit distinct differences in body composition compared to individuals without osteoporosis. In particular, the strong association between visceral fat and senile osteoporosis highlights a potential role for BIA in early risk detection and the development of tailored therapeutic strategies.

## 1 Introduction

Osteoporosis is the most common musculoskeletal disease worldwide. It is characterized by low bone mass and microarchitectural deterioration of bone tissue, increasing the risk of fractures. Osteoporotic fractures are associated with a high mortality rate and represent a considerable health burden. After a fragility fracture of the hip or spine, a 1-year mortality rate of 15 % in women and 22 % in men is assumed. ^1^^,^^2^ There are several factors affecting the development of osteoporosis, including age, female menopause, smoking, exercise, diet and obesity.

Obesity is a significant public health issue in modern society with a continuously increasing prevalence. It is associated with altered metabolic parameters and a chronic inflammatory state. ^3^ The relationship between overweight and osteoporosis is complex and remains not fully understood. While a protective effect of a higher BMI was previously assumed, more recent studies also indicate an increased risk of fractures. This risk appears to depend heavily on the type of adipose tissue and varies across different skeletal sites. 4, 5, 6, 7

Two types of adipose tissue can be distinguished: subcutaneous fat and visceral fat. These differ significantly in structure and function. Visceral fat is generally considered to be associated with an undesirable metabolic status, as seen in conditions such as diabetes and cardiovascular diseases. ^8^ Regarding bone metabolism, visceral fat is increasingly attributed a negative effect. 9, 10, 11, 12, 13, 14 However, an analysis of the Framingham Offspring Cohort also showed an increase in bone mineral density (BMD) with higher amounts of visceral fat. This association, however, was no longer significant after adjusting for BMI. ^15^

Visceral fat is associated with the development of subclinical chronic inflammation. ^16^ It produces adipokines, which appear to play a significant role in bone metabolism. ^17^^,^^18^ Additionally, pro-inflammatory cytokines such as IL-1, IL-6, and TNF-α are produced, which trigger a systemic inflammatory response and negatively impact bone metabolism. 19, 20, 21, 22, 23

Since "osteoimmunology" emerged as a distinct field in 2000, the interplay between inflammatory processes and bone metabolism has received growing attention. ^24^^,^^25^ It is now widely recognized that inflammation accelerates bone resorption. Chronic low-grade inflammation linked to aging, known as "inflammaging”, increased postmenopausal inflammation and systemic inflammatory diseases like rheumatoid arthritis, intensifies bone loss and drives the progression of osteoporosis. ^26^^,^^27^

In clinical practice, different forms of osteoporosis are distinguished. Postmenopausal, senile, and osteoporosis caused by inflammatory rheumatic diseases are among the most significant types.

The postmenopausal form is by far the most common type of osteoporosis. It occurs due to a decline in natural estrogen production in the ovaries following menopause. Estrogens are crucial regulators of bone metabolism, acting as suppressors of RANKL and enhancing the production of OPG in osteoblasts. Additionally, estrogen inhibits the secretion of cytokines such as IL-1, IL-6, and TNFα. The cessation of estrogen production after menopause results in the loss of these positive effects, leading to a negative bone metabolism balance. ^28^

In senile osteoporosis, an imbalance between bone formation and bone resorption arises from the age-related decline in the differentiation capacity of bone-forming cells. Furthermore, chronic inflammation linked to immunosenescence, a condition that progresses with aging, seems to play a pivotal role. ^29^^,^^30^

Patients with inflammatory rheumatic diseases often develop secondary osteoporosis during the course of their illness, regardless of glucocorticoid therapy. Increased release of pro-inflammatory cytokines (particularly TNFα, IL-1, IL-6, and IL-17) by inflammatory cells promotes osteoclastogenesis and leads to bone mass loss in rheumatic patients. ^31^

So far, patients with osteoporosis are treated based on their risk profiles. Diagnostics or therapies tailored to these subtypes are not yet feasible.

Currently, the basic diagnostic process for osteoporosis focuses on taking a medical history to assess risk factors, performing laboratory tests to rule out secondary causes, and measuring bone density using DXA or CT scans. However, these methods are not yet widely available due to the high cost of equipment and the space required.

Recently, the measurement of body composition (BC) using bioelectrical impedance analysis (BIA) has gained increasing importance. This method allows for the relatively inexpensive and non-invasive determination of BC using a portable device. A weak alternating current is used to measure body impedance, from which conclusions about BC are drawn. ^32^

Recent studies highlight the relevance of BIA measurements in assessing osteoporosis risk profiles. ^16^ However, it remains unclear which parameter changes are clinically relevant, and thus BIA measurement has not yet been routinely established in the diagnostic process for osteoporosis patients.

In previous studies, the influence of body composition, particularly visceral fat, on fracture risk has been investigated primarily in young, healthy adults or postmenopausal women. To date, no comparative studies have been conducted on different forms of osteoporosis, such as senile, postmenopausal, and rheumatoid-induced osteoporosis. Moreover, prior research has not focused on a cohort with existing osteoporotic fractures, such as fragility fractures of the hip, which we believe are highly relevant for risk stratification.

Our hypothesis was that body composition, particularly chronic inflammation caused by visceral fat, has a significant impact on the likelihood of osteoporotic fractures and that this effect varies among different forms of osteoporosis. To test this, we prospectively performed BIA measurements on patients with osteoporotic hip fractures and categorized them into different groups based on demographic and clinical characteristics.

## 2 Material and methods

### 2.1 Study design and study population

In this study, 47 patients were prospectively enrolled between November 2022 and November 2024. These patients were treated at the University Hospital Düsseldorf due to osteoporotic fractures of the hip (pertrochanteric femur fracture or femoral neck fracture), fragility fractures of the pelvis, or coxarthrosis. Only women over the age of 60 were included to ensure a better comparison with the postmenopausal osteoporosis group. Exclusion criteria included inability to provide consent, inability to provide medical information, male gender, muscular disorders, and the presence of a pacemaker.

These patients were divided into three groups based on age and the presence of osteoporotic fractures (defined as pertrochanteric femur, femoral neck, or pelvic fractures resulting from a low-energy fall at walking speed or less).

1) The senile osteoporosis group included patients ≥80 years (ages 80–96, mean age 87.4, n = 20) with osteoporotic fractures.
2) The postmenopausal osteoporosis group included patients ≤75 years (ages 61–75, mean age 68.8, n = 14) with osteoporotic fractures.
3) The control group consisted of patients ≤75 years with coxarthritis and without osteoporotic fractures or other osteoporosis-specific risk factors (e.g., history of insufficiency fractures, presence of rheumatic diseases, glucocorticoid therapy; ages 61–75, mean age 69.0, n = 13).

A complete clinical history and examination were conducted upon inpatient admission, focusing on risk factors for osteoporosis, as determined by the guidelines of the German Osteoporosis Society (DVO). Particular attention was given to endocrine diseases, rheumatological diseases, musculoskeletal disorders, cardiovascular diseases, cancer, and fall-associated/geriatric risk factors.

Preoperatively, routine laboratory tests as well as a basic osteological laboratory workup were conducted. The analysis focused on parameters such as serum calcium (mmol/L), serum phosphate (mmol/L), alkaline phosphatase (U/L), parathyroid hormone (PTH) (pmol/L), 25-hydroxyvitamin D3 (ng/mL), hemoglobin (Hb) (g/dL), C-reactive protein (CRP) (mg/dL), and thyroid-stimulating hormone (TSH) (μU/mL).

During the inpatient stay, bioelectrical impedance analysis (BIA) was performed. Additionally, in randomly selected patients from each group, bone mineral density (BMD) measurements using dual-energy X-ray absorptiometry (DXA) were carried out to confirm the group classification based on the risk profile using BMD values. Before BIA measurement, informed consent was obtained, and patients were informed about study participation and data usage.

### 2.2 Body composition measurement

Body composition (BC) was assessed using bioelectrical impedance analysis (BIA) with an InBody S10 device (InBody Europe, Eschborn, Germany). Measurements were conducted on lying, clothed patients with electrodes attached to both wrists and above both ankles. The device recorded bioelectrical impedances, combined with variables such as age, weight, height, and gender. The software calculated parameters such as total body water (L), body fat mass (kg), lean body mass (kg), soft lean mass (kg), fat-free mass (kg), bone mineral content (kg), visceral fat area (cm^2^) and body mass index (BMI) (kg/m^2^).

### 2.3 Geriatric assessment

During the inpatient treatment, a comprehensive geriatric assessment was conducted, which included measuring grip strength, Barthel Index, Katz Index, IADL, DEMMI, SARC-F, CFS, and the upper arm-to-calf circumference ratio.

Handgrip strength was measured using an electronic dynamometer to assess the maximum strength of the right and left hands. Hand strength reflects overall muscle condition, which impacts mobility. Values below 28 kg for men and 18 kg for women indicate reduced grip strength.

The Barthel Index assesses basic daily functions such as eating, bathing, personal hygiene, dressing, bowel and bladder control, toileting, transferring from bed or chair, mobility, and stair climbing. Scores range from 0 to 100, with higher scores indicating greater independence and lower care needs.

The Katz Index evaluates independence in six functional areas: bathing, dressing, toileting, transferring, continence, and feeding. A score of 1 indicates independence, while 0 signifies dependency on supervision, assistance, or comprehensive care. A total score of ≥5 indicates independence, while <5 indicates dependence.

Instrumental Activities of Daily Living (IADL) were assessed using the Lawton IADL scale, covering eight areas: telephone use, shopping, meal preparation, housekeeping, laundry, transportation, medication management, and financial management. Each item is scored 0 (requires assistance) or 1 (independent), with a maximum score of 8 indicating complete independence.

The De Morton Mobility Index (DEMMI) assesses the mobility of geriatric patients through 15 items across five categories (bed, chair, static balance, walking, and dynamic balance). Points are added to calculate a raw score, converted into a DEMMI score. The categories are: very limited mobility (DEMMI = 0–24), limited mobility (DEMMI = 27–39), moderately restricted mobility (DEMMI = 14–57), and independent mobility (DEMMI = 62–100).

### 2.4 Bone density measurement

Bone mineral density (BMD) was determined via DXA measurement (Lunar prodigy, GE HealthCare GmbH, Chicago, USA). According to the World Health Organization (WHO), osteoporosis is diagnosed when bone mineral density at the lumbar spine and/or proximal femur deviates by more than 2.5 standard deviations (T < -2.5) from the mean of a reference population.

### 2.5 Sarcopenia screening

Sarcopenia was screened using the SARC-F score, a questionnaire with five items: strength (S), assistance with walking (A), rising from a chair (R), climbing stairs (C), and falls (F). Each category was rated from 0 (no difficulty) to 2 (severe difficulty), with a total score of ≥4 indicating sarcopenia. Muscle mass was measured using bioelectrical impedance analysis.

### 2.6 Frailty screening

Frailty syndrome was diagnosed using the Clinical Frailty Scale (CFS), which evaluates comorbidity, function, and cognition to assign a frailty score from 1 (very fit) to 9 (terminally ill).

### 2.7 Ethical approval

This study was conducted with the approval of the Ethics Committee of the Medical Faculty at Heinrich Heine University Düsseldorf (Study No. 2021–1412, Amendment Study No. 2021–1412_1, March 19th^,^ 2023) and in accordance with the declaration of Helsinki. Informed consent was obtained for experimentation with human subjects.

### 2.8 Statistics

Data acquisition and analysis was performed using the GraphPad PRISM8 (Boston, MA,USA) and Excel (Microsoft, Redmond, WA, USA) software. Data are expressed as mean ± standard deviation. Mann-Whitney-U and Kruskal-Wallis tests were applied to compare statistical significance of the results. Holm-Sidak and Dunn's methods were used to correct for multiple comparisons. P-values lower than 0.05 were considered statistically significant.

## 3 Results

### 3.1 Patient characteristics

In the senile osteoporosis group (n = 20), 15 patients (75 %) presented with a hip fracture, while 5 (25 %) had a pelvic fracture. In the postmenopausal osteoporosis group, 9 patients (75 %) presented with a hip fracture, and 3 (25 %) with a pelvic fracture. Notably, 50 % of patients with pelvic fractures underwent surgical treatment with sacral screws, classified as FFPIIc. Among conservatively treated patients, two had FFPIa fractures and two had FFPIIb fractures.

Patients in the senile osteoporosis group exhibited significantly greater frailty, as measured by the Clinical Frailty Scale (CFS; mean 4.1, SD 1.5), compared to the control group (mean 2.2, SD 0.4, p < 0.001). The postmenopausal group displayed intermediate frailty (mean 3.4, SD 1.2; p = 0.025 vs. control). No statistically significant difference was observed between the two osteoporosis groups.

Geriatric assessment data were available for the senile group and 7 patients (50 %) of the postmenopausal group, but not for the control group. The senile osteoporosis group demonstrated significantly higher levels of sarcopenia, as measured by the SARC-F (mean 4.6, SD 2.0) and lower grip strength (mean 16.4, SD 9.6) compared to the postmenopausal group (sarcopenia: mean 2.5, SD 1.6, p = 0.005; grip strength: mean 21.6, SD 7.8, p = 0.303, Table 1).

### 3.2 Laboratory findings

Routine and osteological laboratory test results are summarized in Table 2. Vitamin D3 levels were significantly lower in the senile group compared to the control group (17.2 vs. 45.0, p = 0.019). In the postmenopausal group, Vitamin D3 levels were also reduced (21.6 vs. control), though not significantly (p = 0.519). The senile group exhibited a higher, albeit narrowly not statistically significant, prevalence of latent hypothyroidism compared to the control group (TSH 2.4 vs. 1.0, p = 0.05).

**Table 1:** Patient characteristics.

	senile (n = 20)	postmenopausal (n = 7)	P-values
Grip strength (kg)	16.4 (9.6)	21.6 (7.8)	0.303
SARC-F	4.6 (2.0)	2.5 (1.6)	0.005
hip fracture	15	9	–
pelvis fractur	5	5	–

Data are indicated as mean with standard deviation. Hip fracture refers to pertrochanteric or femoral neck fracture (see Table 1).

**Table 2:** Laboratory examinations.

	senile (n = 20)	postmenopausal (n = 14)	control (n = 13)
calcium (mmol/l)	2.4 (0.3)	2.3 (0.4)	2.4 (0.5)
phosphate (U/l)	1.1 (0.3)	0.9 (0.2)	1.2 (0.4)
alkaline phosphatase (U/l)	74.6 (25.2)	84.5 (39.9)	80.2 (57.5)
Vit. D3 (ng/ml)	16.4 (13.7)	21.6 (13.5)	45.0 (51.0)
PTH (pmol/l)	7.7 (4.8)	6.1 (2.8)	6.3 (4.3)
Hb (g/dl)	11.6 (2.2)	11.0 (2.5)	10.7 (1.3)
CRP (mg/dl)	2.6 (3.5)	3.8 (2.8)	3.1 (1.8)
TSH (μU/ml)	2.4 (2.2)	1.6 (0.9)	1.0 (0.6)

Data are indicated as mean with standard deviation. Vit. D3: 25-Hydroxyvitamin D3; PTH: Parathyroid hormone; Hb: Hemoglobin; CRP: C-reactive protein; TSH: Thyroid-stimulating hormone.

### 3.3 Bioelectrical impedance analysis (BIA)

**Fig. 1:**
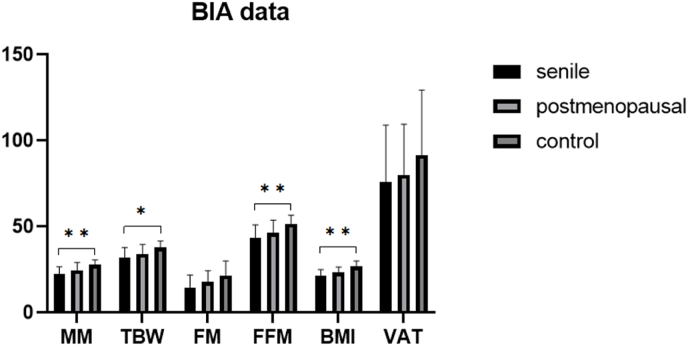
Bioelectrical Impedance Analysis of the study cohort. MM: Muscle Mass; TBW: Total Body Water, FM: Fat Mass, FFM: Fat-free Mass; BMI Body Mass Index; VAT: Visceral Adipose Tissue. Data are presented as mean with standard deviation. ∗p < 0.05, ∗∗p < 0.01, ∗∗∗p < 0.001.

To assess body composition, BIA measurements were performed, evaluating muscle mass (MM), total body water (TBW), fat mass (FM), fat-free mass (FFM), BMI, visceral adipose tissue (VAT), and bone mineral content (BMC) (Fig. 1).

Muscle mass was significantly reduced in the senile group compared to controls (22.5 vs. 27.7, p = 0.002). The postmenopausal group exhibited intermediate values (24.5), not reaching statistical significance (p = 0.164). Similar trends were observed for TBW (senile: 32.0, postmenopausal: 34.0, control: 37.9; p = 0.012 for senile vs. control) and FFM (senile: 43.4, postmenopausal: 46.2, control: 51.6; p = 0.008 for senile vs. control), with no significant differences between the osteoporosis groups.

Fat mass also followed this pattern (senile: 14.4, postmenopausal: 17.6, control: 21.6) without significant intergroup differences. The BMI was significantly lower in the senile group compared to controls (21.6 vs. 26.6; p = 0.001), while the postmenopausal group had intermediate values (23.3).

**Fig. 2:**
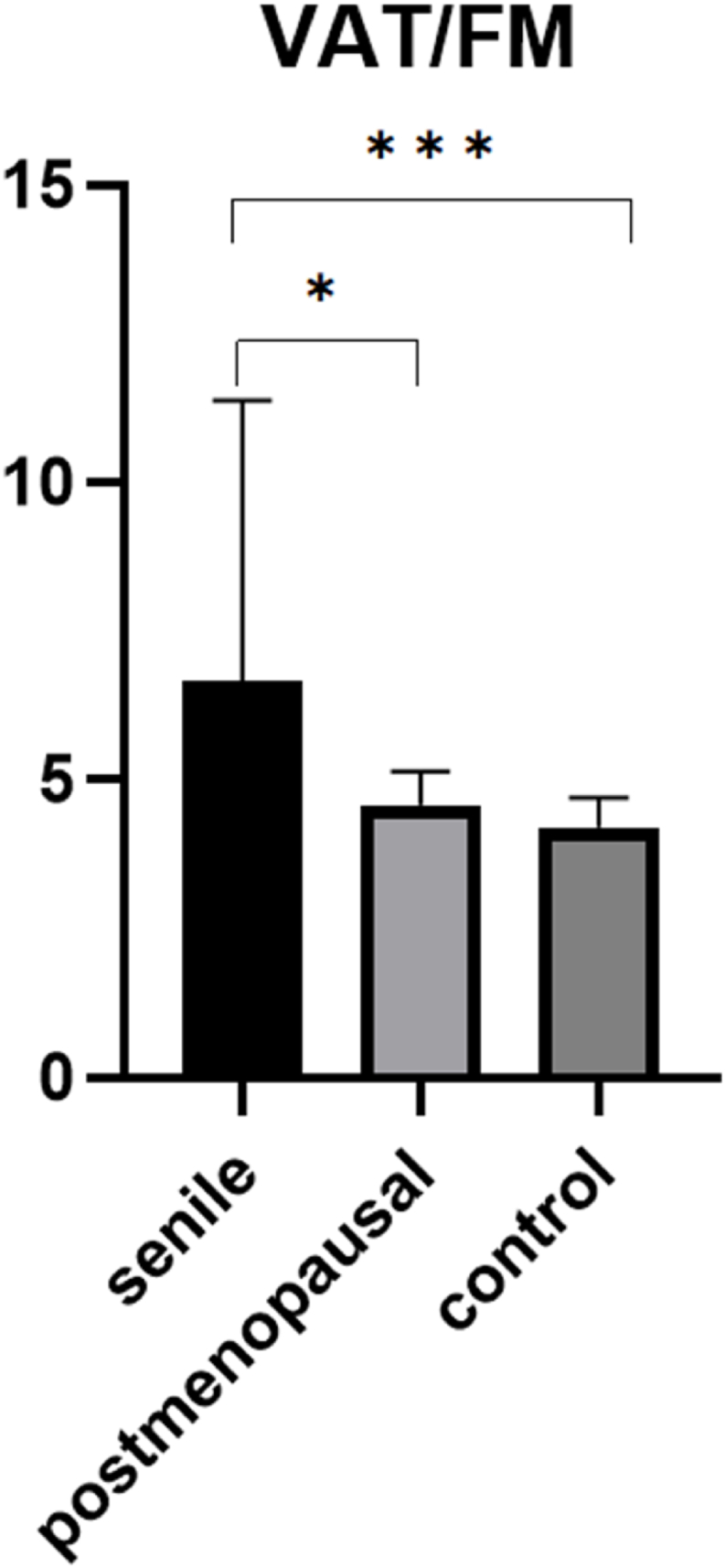
VAT/FM ratio of the three study groups. VAT: Visceral Adipose Tissue; FM: Fat Mass. Data are presented as mean with standard deviation.

VAT showed no significant differences between the groups. However, as expected, VAT increased with overall fat mass. To determine the relative proportion of VAT, we calculated a ratio (VAT/FM; see Fig. 2). This revealed a significantly higher relative proportion of visceral fat in the senile group compared to the control group (6.7 vs. 4.2; p < 0.001). The postmenopausal group showed a higher ratio than the control group (4.6), though this difference was not statistically significant, likely due to the small sample size. Of note, a significant difference between the osteoporosis groups could be observed (p = 0.016). This indicates that the higher relative proportion of visceral fat in senile as well as postmenopausal osteoporosis patients could indeed have an impact on the increased fracture risk in these patients.

### 3.4 Bone mineral content (BMC) and DEXA measurements

**Fig. 3:**
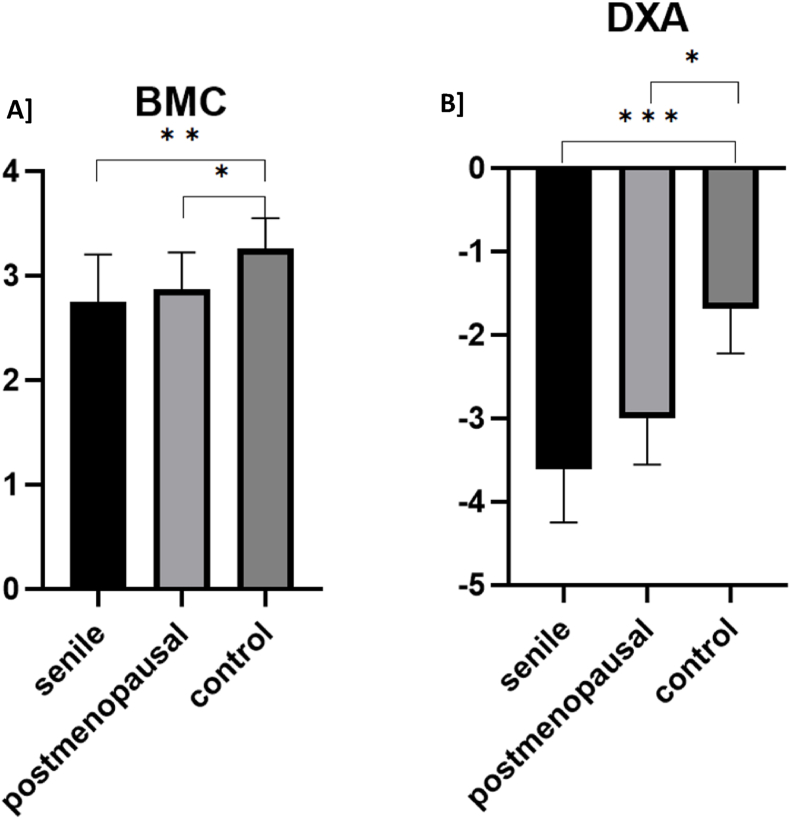
Bone Mineral Content and Dual-Energy X-ray absorptiometry of the three subgroups. A] The BMC (in kg) in respective groups is shown. B] The results of the DXA measurements are presented. The T-score indicates the standard deviation compared to a healthy reference cohort. BMC: Bone Mineral Content; DXA: Dual-energy X-rax absorptiometry; ns: non-significant; ∗∗: p < 0.001; ∗∗∗: p < 0.0001. Data are presented as mean with standard deviation.

BMC was significantly lower in both osteoporosis groups compared to the control group (senile: 2.7 kg, postmenopausal: 2.9 kg, control: 3.3 kg; p = 0.003 and p = 0.048). DEXA measurements indicated significantly lower mean t-values in the senile group (−3.5) compared to controls (−1.7, p < 0.001), while the postmenopausal group had intermediate values (−3.0; p = 0.042 vs. control). No significant differences were found between the two osteoporosis groups (Fig. 3A and B).

These results confirm that both osteoporosis groups not only differ in age and fracture type but also exhibit osteoporotic bone mineral density, consistent with the classification.

## 4 Discussion

In this study, we demonstrated for the first time that senile and postmenopausal osteoporosis patients differ in body composition and both exhibit a relatively higher proportion of visceral fat compared to non-osteoporotic patients.

The postmenopausal and control groups were approximately the same age; however, the postmenopausal group was significantly more frail on average. Frailty in old age is known to have a strong association with the occurrence of osteoporosis. ^33^ This is also confirmed in our population, with a CFS of 4.5 in the senile group. The relationship between frailty and osteoporosis in younger postmenopausal women is less well known. In our opinion, this connection warrants further investigation to evaluate the feasibility of screening for frailty in postmenopausal osteoporosis patients and, conversely, osteoporosis screening in younger patients with frailty syndrome. Our data also confirm the well-known correlation between vitamin D levels and bone density. ^34^

Both muscle mass measured by BIA and the SARC-F score were reduced in the senile and postmenopausal groups. In the senile osteoporosis group, and to a lesser extent in the postmenopausal group, BMI, FM, TBW and FFM were also reduced. This aligns with frailty and sarcopenia, suggesting a general catabolic metabolism and associated functional impairments in these osteoporosis patients. As recently demonstrated by Sgarro and colleagues in a cluster analysis, a reduction in TBW, FFM, and MM could have predictive value for the development of osteoporosis. ^16^ Their study analyzed young overweight patients aged 35, in whom, unlike in our data, fat mass was elevated. In this and other studies, a negative correlation between fat mass and BMD was observed. ^35^^,^^36^ Our data on the other hand indicate that fat mass is reduced in cases of manifest osteoporosis.

Total visceral adipose tissue was also reduced in the osteoporosis groups, which was to be expected with the declined amount of total body fat in osteoporosis patients. We therefore calculated visceral fat in relation to total body fat to determine the relative proportion of visceral fat. Interestingly, both the senile and postmenopausal groups exhibited a higher relative proportion of visceral fat. However, the postmenopausal group narrowly missed statistical significance in comparison to the control group, likely due to small sample sizes. It can be postulated that while fat mass appears to have a positive predictive value for the onset of osteoporosis, it is reduced in cases of manifest osteoporosis. At the same time, the relative proportion of visceral fat seems to increase. This mechanism appears to be particularly pronounced in patients with senile osteoporosis, potentially playing a role in the progression of the disease. But even in young patients, increased VAT appears to have a massive impact on bone metabolism. To this extend, the study by Sharma and colleagues recently demonstrated the negative predictive value of increased VAT (adjusted for BMI) regarding bone density and the bone metabolism parameters CTX-1 (C-terminal telopeptide of type 1 collagen) and osteocalcin in young, overweight, non-osteoporotic patients. ^37^

Three mechanisms are postulated through which adipose tissue can influence bone metabolism.

1) Endocrine effects via the secretion of cytokines by adipocytes, 2) Adipokines that affect bone metabolism through modulation of the central nervous system, and 3) Paracrine effects of adipocytes within the bone marrow. ^38^ Adipocytes, particularly those in visceral fat, can secrete cytokines such as IL-1, IL-6, and TNF-α, thereby promoting bone resorption. ^23^ Adipokines such as adiponectin and leptin can influence osteoblasts and osteoclasts directly, as well as modulate sympathetic tone by acting on the hypothalamus. Increased sympathetic activity can mediate bone resorption by upregulating RANKL.^39^ Additionally, adipocytes in the bone marrow niche can exert paracrine effects on the differentiation of MSCs, leading to a shift toward increased adipocyte formation and reduced osteoblast differentiation. ^40^

Different forms of osteoporosis are distinguished, each with fundamentally different pathophysiological mechanisms.

The postmenopausal form arises due to a decline in natural estrogen production in the ovaries after menopause. Estrogens are significant regulators of bone metabolism. Among other effects, they act as suppressors of RANKL and enhance the production of Osteoprotegerin (OPG) in osteoblasts. Estrogen also inhibits the secretion of cytokines such as IL-1, IL-6, and TNFα. A cessation of estrogen production after menopause leads to the loss of these positive effects, resulting in a negative balance in bone metabolism. ^41^^,^^42^

Senile osteoporosis, on the other hand, is characterized by chronic subclinical inflammation, increased adipogenesis, reduced osteogenesis, and enhanced production of reactive oxygen species (ROS), all of which contribute to increased bone resorption. ^30^

To date, no subtype-specific therapy or early detection is available. In our study, we demonstrated significant differences in the extent of visceral fat and between these patient groups. These finding may contribute to the future establishment of individualized clinical management for different osteoporosis patients.

We acknowledge, that this study is not without limitations. The classification into different subgroups was based on age and the presence of osteoporotic fractures. Randomly selected DXA measurements within the respective subgroups confirmed osteoporotic bone density values in the osteoporotic groups. However, this method of classification does not rule out a certain bias, and future studies should adopt a stricter classification system based on the timing of menopause and precise bone density measurements. Moreover, the relatively small sample size limited the statistical power of the study, with some results showing clear trends but failing to reach statistical significance, especially in the postmenopausal group.

In summary, this study demonstrated that patients with senile and postmenopausal osteoporosis differ in body composition compared to non-osteoporotic patients. Visceral fat, in particular, appears to be elevated in senile patients and to a lesser extent in postmenopausal patients. It is well known that visceral fat can produce cytokines and trigger inflammatory responses.

These findings suggest that body composition, particularly visceral fat, may play a significant role in the development and maintenance of senile osteoporosis and, to a lesser extent, postmenopausal osteoporosis.

Future large prospective studies should determine the actual cytokine production of visceral fat in different osteoporosis subtypes and assess the effect these cytokines have on bone metabolism in the respective patient cohort. This could potentially lead to measures for early detection of developing osteoporosis by BIA measurements and the exploration of new, patient-specific therapeutic approaches.

## CRediT authorship contribution statement

**N. Wergen:** Conceptualization, Data curation, Investigation, Methodology, Validation, Writing – original draft. **U. Maus:** Conceptualization, Formal analysis, Funding acquisition, Methodology, Supervision, Validation, Writing – review & editing. **K. Schultz:** Formal analysis, Validation, Writing – review & editing. **H. Frohnhofen:** Data curation, Methodology, Validation, Writing – review & editing. **D. Latz:** Formal analysis, Validation, Writing – review & editing. **C. Somsen:** Data curation, Investigation, Methodology, Validation, Writing – review & editing. **L. Mueller:** Data curation, Investigation, Methodology, Validation, Writing – review & editing. **C. Beyersdorf:** Conceptualization, Data curation, Funding acquisition, Investigation, Methodology, Project administration, Software, Supervision, Validation, Visualization, Writing – original draft, Writing – review & editing.

## Informed consent

Written informed consent was obtained from all individual participants (or their legal guardians) included in the study.

## Ethical statement

This study was conducted with the approval of the Ethics Committee of the Medical Faculty at Heinrich Heine University Düsseldorf (Study No. 2021–1412, Amendment Study No. 2021–1412_1, March 19th^,^ 2023) and in accordance with the declaration of Helsinki.

## Funding

This study is funded by the Paul-Kuth-Stiftung. CB is funded by the FUTURE program of the German Research Foundation (Deutsche Forschungsgemeinschaft, DFG))–493659010.

## Conflict of interest

All authors declare, that they have no conflict of interest.
